# Riboflavin for COVID-19 Adjuvant Treatment in Patients With Mental Health Disorders: Observational Study

**DOI:** 10.3389/fphar.2022.755745

**Published:** 2022-03-10

**Authors:** R. A. Akasov, E. V. Khaydukov, D. S. Andreyuk, N. V. Sholina, A. N. Sheremeta, D. V. Romanov, G. P. Kostyuk, V. Ya. Panchenko, M. V. Kovalchuk

**Affiliations:** ^1^ Federal Scientific Research Center Crystallography and Photonics Russian Academy of Sciences, Moscow, Russia; ^2^ I.M. Sechenov First Moscow State Medical University, Moscow, Russia; ^3^ Alekseev Psychiatric Clinical Hospital, Moscow, Russia; ^4^ Lomonosov Moscow State University, Moscow, Russia; ^5^ NRC «Kurchatov Institute», Moscow, Russia

**Keywords:** SARS-CoV-2, COVID-19, riboflavin, flavin mononucleotide, inflammation, cytokines, schizophrenia, organic mental disorders

## Abstract

**Background:** COVID-19 treatment remains a challenge for medicine because of the extremely short time for clinical studies of drug candidates, so the drug repurposing strategy, which implies the use of well-known and safe substances, is a promising approach.

**Objective:** We present the results of an observational clinical study that focused on the influence of riboflavin (vitamin B2) supplementation on the immune markers of COVID-19 severity in patients with mental health disorders.

**Results:** We have found that 10 mg of flavin mononucleotide (a soluble form of riboflavin) intramuscularly twice a day within 7 days correlated with the normalization of clinically relevant immune markers (neutrophils and lymphocytes counts, as well as their ratio) in COVID-19 patients. Additionally, we demonstrated that total leucocytes, neutrophils, and lymphocytes counts, as well as the neutrophils to leucocytes ratio (NLR), correlated with the severity of the disease. We also found that patients with organic disorders (F0 in ICD-10) demonstrated higher inflammation then patients with schizophrenia (F2 in ICD-10).

**Conclusion:** We suggest that riboflavin supplementation could be promising for decreasing inflammation in COVID-19, and further evaluation is required.

This observational clinical trial has been registered by the Sverzhevsky Research Institute of Clinical Otorhinolaryngology (Moscow, Russia), Protocol No. 4 dated 05/27/2020.

## Introduction

COVID-19 is a new and fast-growing challenge for medicine all over the world. Currently, there is a lack of evidence concerning the drugs with proved clinical efficacy against COVID-19 due to the limited time for laboratory and clinical evaluations. In this case, a drug repurposing strategy that involves the screening of existing compound libraries could be promising. The COVID-19 treatment used today is supportive, and the main cause of death is associated with respiratory failure due to acute respiratory distress syndrome (ARDS) (Mehta et al., 2020; Dubina, 2022). It is believed that one of the main causes of ARDS is the so-called “cytokine storm” (Giamarellos-Bourboulis et al., 2020), at which extremely high levels of inflammation markers in plasma are observed, including C-reactive protein and pro-inflammatory cytokines (IL-6, TNFα, IL-8, IL-2, etc).

Riboflavin (Rf), also called vitamin B2, is a precursor of essential coenzymes such as flavin mononucleotide (FMN) and flavin adenine dinucleotide (FAD), which play a vital role in cellular metabolism and have been demonstrated as promising anti-inflammatory and anti-oxidative agents (Thakur et al., 2017; Ahn and Lee, 2020; Suwannasom et al., 2020). Rf (0.2 mg/kg, i. p., single dose) protected against acute oxidant-mediated inflammatory injury in the lungs of Long-Evans rats (Seekamp et al., 1999). FAD significantly decreased inflammatory cell infiltration, reduced lung injury scores, and ameliorated lung edema in a mice model of influenza A H5N1 virus-induced lung injury (Huang et al., 2020). Rf supplementation (25 mg/kg/d, 3 days) prevents abdominal aortic aneurysm formation in a rat model through an antioxidant effect of endogenous superoxide dismutase activation (Yu et al., 2016). Diabetic mice which received Rf (10 or 20 mg/kg/day, p. o.) demonstrated the decrease of oxidative stress with an increased glucose uptake in skeletal muscles and white adipose tissue. Histological studies showed recovery in the liver and kidney tissue injury (Alam et al., 2015). More importantly, the efficacy of a riboflavin-based strategy in relieving inflammation and oxidative stress has been demonstrated in several recent clinical trials. In a double-blind, phase IIb clinical trial, patients with suspected stroke of less than 3 h of evolution received a single intravenous administration of 20 mg of Rf (da Silva-Candal et al., 2018). The decrease in glutamate concentration was significantly greater in the Rf-treated group. The percentage improvement according to the National Institutes of Health Stroke Scale score was higher in the Rf-treated group than in the placebo one (da Silva-Candal et al., 2018). Rf supplementation in patients with Crohn’s disease, a type of inflammatory bowel disease (IBD), has been evaluated in a recent prospective clinical intervention study (von Martels et al., 2020). Patients received 100 mg Rf daily for 3 weeks, which resulted in a reduction in systemic oxidative stress and anti-inflammatory effects. The concentration of free thiols significantly increased, while the concentration of IL-2 significantly decreased after 3 weeks. Serum C-reactive protein concentration also decreased after Rf supplementation, but in the subgroup with high fecal calprotectin levels only, which is usually discussed as aа marker of active inflammation. TNF-α also decreased in this group. Rf supplementation (10 mg/day, p. o) significantly decreased plasma homocysteine, a marker of inflammation and ischemic injury, in the group of elderly people with low Rf status (Tavares et al., 2009). It should be noted, that Cytoflavin (Inosine + Nicotinamide + Riboflavin + Succinic Acid) has been recently proposed for post-COVID syndrome treatment, and an anti-asthenic effect, correction of cognitive impairments, and a decrease in the severity of thrombocytopenia have been demonstrated (Putilina et al., 2021). Recently, we also discussed Rf-associated pathways as a possible target to suppress secondary infections at COVID-19 *via* the mucosal-associated invariant T cells activity (Akasov and Khaydukov, 2020). Several in silico studies proposed Rf/FMN/FAD as possible antiviral compounds potentially able to inhibit papain-like proteinase (PLpro) and 3C-like main protease (3CLpro) of SARS-CoV-2 (Wu et al., 2020; Anwaar et al., 2021; Hooshmand et al., 2021). The possible involvement of B vitamins in COVID-19 has also been discussed (Shakoor et al., 2021). Based on all the data discussed above, mainly on the clinically relevant efficacy in both acute (ischemic stroke) and chronic (Crohn’ disease) inflammation, we assumed the benefits of high doses of FMN (>10 mg per day intramuscularly) in COVID-19 therapy.

It is known that people with mental disorders have a higher chance of being infected with COVID-19 (De Picker et al., 2021; Taquet et al., 2021; Wang et al., 2021), and when infected they are at increased risk of a severe (Lee et al., 2020) or fatal course of illness (Nemani et al., 2021). The risk is aggravated not only by behavioral peculiarities, but also by immunological disturbances related to the nature of the mental disorder or associated medical treatment (Maes et al., 2012; Zhou et al., 2021). Moreover, patients with mental disorders often suffer from obesity, diabetes, chronic lung disease, and hypertension (De Hert et al., 2011) which can worsen the course of an illness.

The aim of the current research was to evaluate the immune patterns in COVID-19 patients with mental disorders and evaluate the possible benefits of riboflavin supplementation for COVID-19 treatment in an observational study.

## Materials and Methods

### Study Design and Participants

We recruited 119 symptomatic adult inpatients (76 male, 43 female, mean age 59.3 ± 16.7 years) with mental disorders treated for COVID-19 at Alekseev Psychiatric Clinical Hospital no. 1 (Moscow, Russia) in June–July 2020; patients were at hospital within the treatment course. The inclusion criteria were as follows: (Mehta et al., 2020): at least 18 years of age; (Dubina, 2022); confirmed COVID-19 diagnosis (a positive test for SARS-CoV-2 RNA detected by RT-PCR collected from the upper respiratory tract; or pulmonary radiological data specific for COVID-19; or antibodies ratio specific for acute viral infection). Exclusion criteria: (Mehta et al., 2020): known or suspected active viral, bacterial, mycobacterial, or fungal infection other than COVID-19, including Epstein-Barr virus, cytomegalovirus, herpesvirus family, HIV, hepatitis C virus, etc; (Dubina, 2022); pregnancy and/or breastfeeding; (Giamarellos-Bourboulis et al., 2020); oncology diseases. The participants were assigned to experimental (50 patients) and control (69 patients) groups. All of these patients received antiviral treatment according to the national clinical guideline (Ministry of Health of Rusussian Federation, 2020), namely chloroquine (500 mg twice a day for 7 days), hydroxychloroquine (400 mg twice at the first day, then 200 mg twice at the next 6 days), lopinavir-ritonavir combination (400 mg + 100 mg p. o. every 12 h within 14 days), azithromycin (500 mg p. o, 5 days, in combination with hydroxyloroquine), and interferon preparations (IFN-*β*1b, 0.25 mg/ml, 8,000,000 ME, 14 days; IFN-*α*2b, 3,000 ME, 5 times a day, 5 days). Additionally, dexamethasone (12 mg per day p. o. or 4 mg three times a day i. v.) was used in case of CRP value growth. In addition, patients in the experimental group received the full course of riboflavin supplementation on medical advice (10 mg flavin mononucleotide intramuscularly twice a day).

### Data Collection

Demographic, clinical, treatment, and outcome data were obtained from the electronic medical records of Alekseev Psychiatric Clinical Hospital no. 1 (Moscow, Russia). Data were anonymized by removing personally identifiable information prior to processing. Laboratory data included RBC, platelets, WBC, lymphocytes, neutrophils, and monocytes counts determined using a SYSMEX hematological analyzer (Japan); serum levels of C-reactive protein and hemoglobin; serum levels of IL-1β, IL-2, IL-6, MCP-1, TNF-α, and IFN-*γ* cytokines assessed using ELISA reagent kits (Vector-Best, Novosibirsk, Russia); chest computed tomography (CT) scans and their description by radiologists (unilateral and bilateral ground glass opacity, and lung involvement).

### Statistics

Statistical processing of the results was carried out using the GraphPad Prism software, version 6.01. The *p* values were estimated using the Wilcoxon Matched-Pairs Signed Ranks Test (paired, non-parametric, two-tailed) or Mann–Whitney *U* test (non-paired, non-parametric, two-tailed). The data are presented as median [IQR] values or box-and-whiskers plots using Tukey’s modification.

## Results and Discussion

### Group Characteristics

We analyzed demographic, clinical, treatment, and outcome data for 119 patients with mental disorders treated for COVID-19 at Alekseev Psychiatric Clinical Hospital no. 1 (Moscow, Russia) in June–July 2020. All of these patients received antiviral treatment according to the national clinical guideline (Ministry of Health of Rusussian Federation, 2020). We retrospectively divided this cohort to two groups: the first one additionally received 2 × 10 mg/day FMN intramuscularly (10 mg twice a day) on medical advice within 1 week; the second one did not receive FMN supplementation (Table 1).

**TABLE 1:** General characteristics of studied cohort.

	FMN-adding	No FMN-adding
Total number of patients	50	69
Average age ±SD, years	58.6 ± 15.6	59.8 ± 17.5
Male/female ratio	38/12	38/31
Survival	47/50	67/69
CT grade 1/CT grade 2–4 ratio	35/15	58/11
Median CRP value on admission to the hospital	18.20 [2.570; 64.24]	14.21 [3.285; 37.11]

The groups were comparable in terms of age [median (IQR) 59.00 (48.25; 68.60) for the experimental group vs 62.50 (47.00; 72.75) for the control group, *p* = 0.4192 in Mann–Whitney *U* test), CRP value on admission to the hospital [18.20 (2.570; 64.24) vs. 14.21 (3.285; 37.11), *p* = 0.5331], CT grade 1/CT grade 2–4 ratio (two-tailed *p* value of 0.0761 in Fisher’s exact test), and outcome (two-tailed *p* value of 0.6486 in Fisher’s exact test). The only statistically significant difference between the experimental and control groups is the male/female ratio with a predominance of men in the experimental group (two-tailed *p* value of 0.0214 in Fisher’s exact test).

The distribution of the study sample patients according to ICD-10 diagnoses of mental disorders is listed in Table 2. In total, most patients (*n* = 65; 55.1%) were diagnosed with schizophrenia spectrum disorders, F2 in ICD-10. Different types of organic mental disorders, F0 in ICD-10, accounted for 26.7% of cases. Other disorders, including affective disorders (8.8%) and intellectual disability (2.2%), were less frequent. The distribution of ICD-10 diagnoses within the FMN and control groups can also be considered comparable [F (1,3) = 9.417; *p* = 0.0546 in two-way ANOVA]. Based on these demographical and clinical parameters, we assume that both studied groups are similar in their demographic and clinical characteristics.

**TABLE 2:** Distribution of the patients (*n* = 119) according to ICD-10 diagnoses of mental disorders in the FMN-adding group (*n* = 50) and control group (*n* = 69).

Diagnosis (ICD-10 code)	FMN-adding group, n (%)	Control group, n (%)
Organic mental disorders (F0)	16 (32.0%)	25 (36.3%)
Schizophrenia spectrum disorders (F2)	30 (60.0%)	35 (50.7%)
Affective disorders (F3)	4 (8.0%)	7 (10.1%)
Oligophrenia (F7)	0 (0.0%)	2 (2.9%)

### Correlation of Blood and Biochemical Parameters With COVID-19 Severity

**FIGURE 1:**
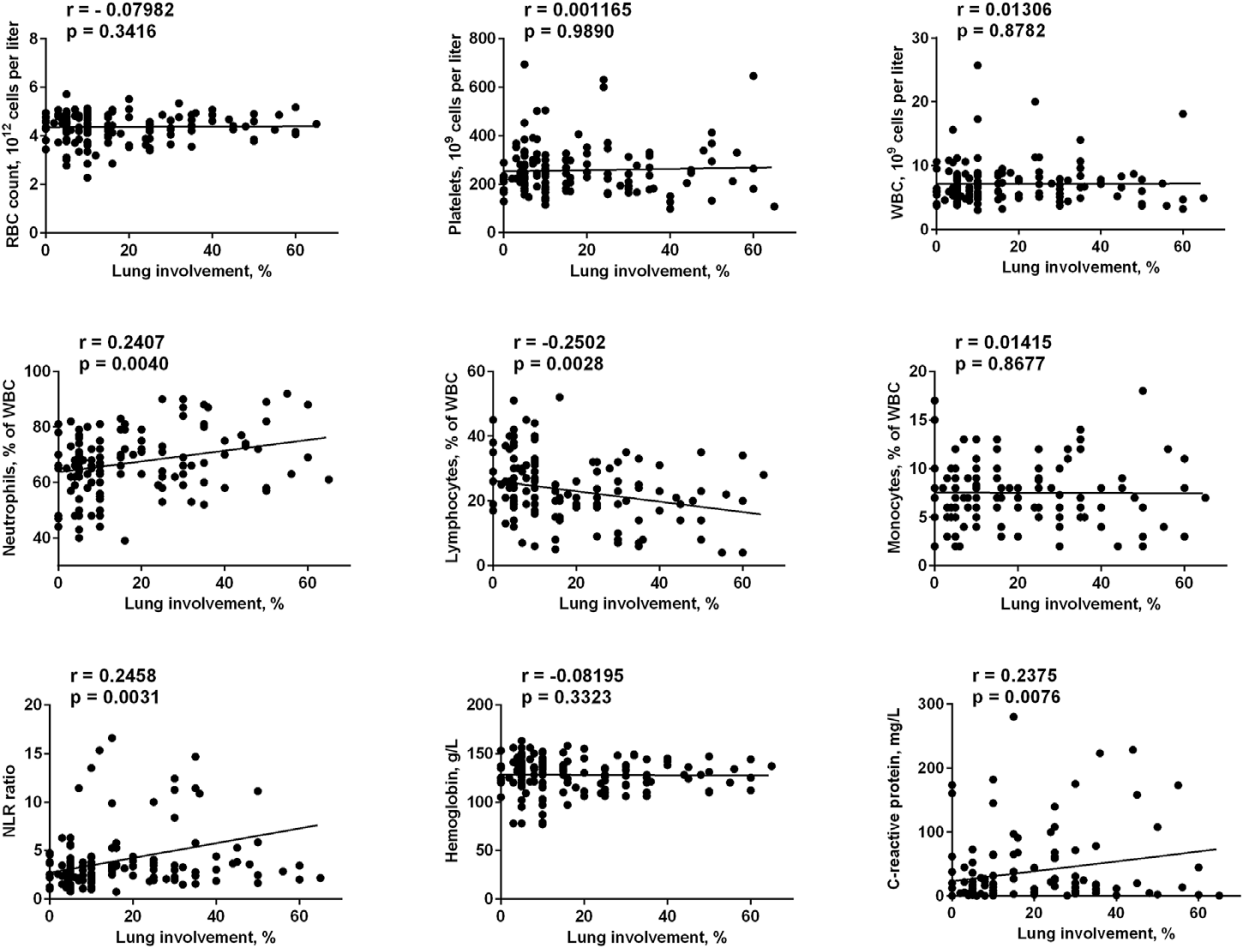
Correlation of main blood and biochemical parameters taken on admission to the hospital with the lung involvement in chest computed tomography. The data on day 1 for FMN group and days 1 and 7 for control group were used.

**FIGURE 2:**
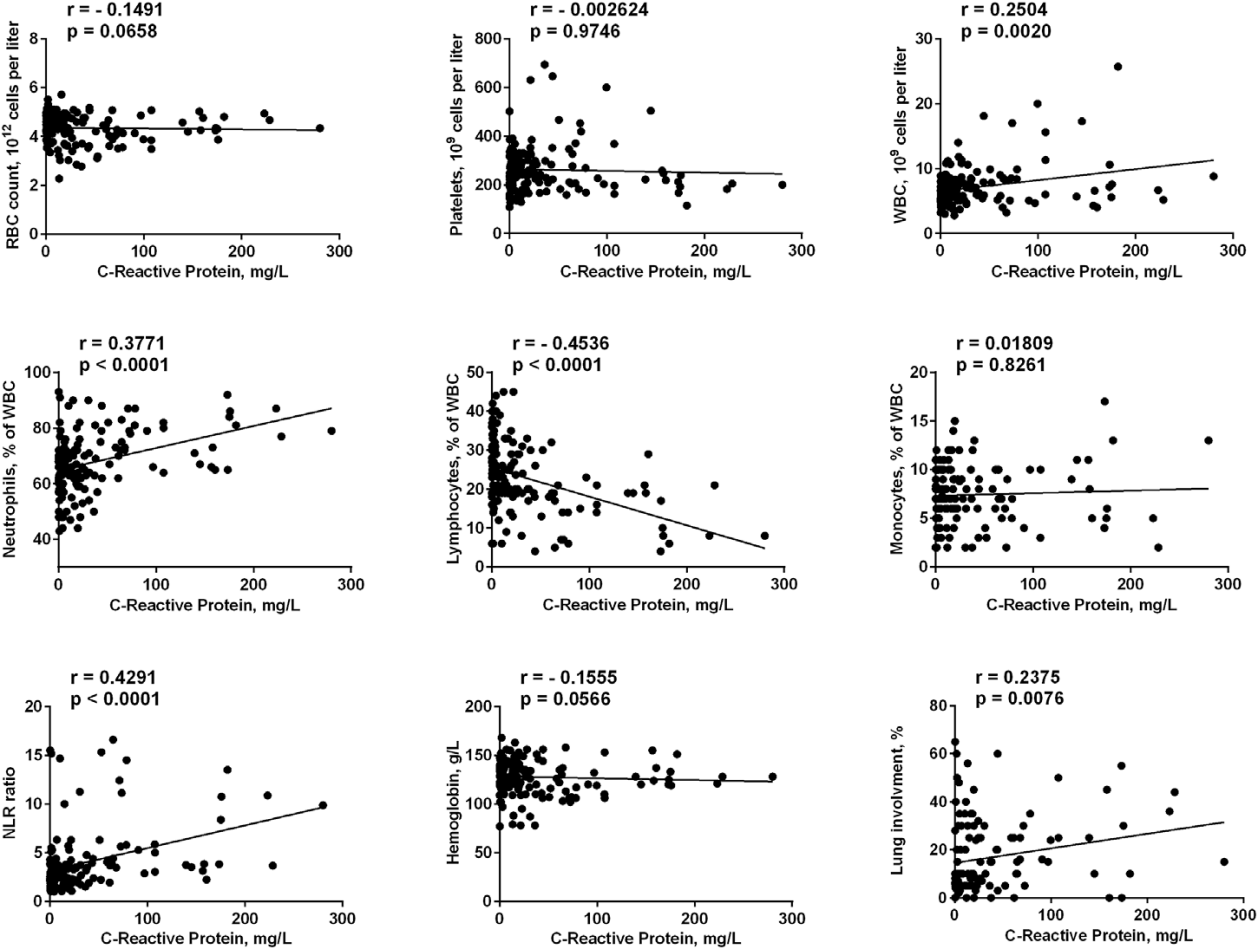
Correlation of main blood and biochemical parameters taken on admission to the hospital with C-reactive protein. The data on day 1 for FMN group and days 1 and 7 for control group were used.

We analyzed a number of the main blood and biochemical parameters, namely RBC, platelets, WBC, lymphocytes, neutrophils, monocytes, and hemoglobin values in both groups. The correlation of these parameters with the two most clinically relevant characteristics (C-reactive protein value and lung involvement in chest computed tomography) was evaluated. It was found that neutrophils values positively correlated with the severity of the disease (with both CRP value and lung involvement), while lymphocytes values correlated with these parameters in a negative manner (Figure 1, 2). Accordingly, the neutrophils to lymphocytes ratio also correlated positively.

The only blood test parameter that correlated with CRP values, but not with the lung involvement, was the total leucocytes count. This could be explained by a better sensitivity of the CRP value to the inflammation status. Indeed, for all studied parameters the correlation with CRP values was clearer than with the lung involvement (e.g., for neutrophils r = 0.3771, *p* < 0.0001 vs. r = 0.2407, *p* = 0.004). Finally, lung involvement positively correlated with CRP values (r = 0.2375, *p* = 0.0076). We can conclude that in our cohort we had four parameters that correlated with the severity of the disease (in addition to well-established clinically relevant lung involvement and CRP values), namely total leucocytes, neutrophils, lymphocytes, and NLR, while RBC, platelets, monocytes and hemoglobin did not correlate with COVID-19 progression. This is in agreement with the literature data: neutrophils and lymphocytes counts, as well as their ratio, were the parameters that predicted the severity and outcome of the COVID-19 infection (Li et al., 2020; Yang et al., 2020; Imran et al., 2021).

### Correlation of Blood and Biochemical Parameters With FMN Supplementation

The median values of the main blood and biochemical parameters, namely RBC, platelets, WBC, lymphocytes, neutrophils, monocytes, hemoglobin, and CRP, were estimated and compared in each group (day 1 vs. day 7) and in each time point (experimental group vs. control group), and summarized in Table 3. Neutrophils and lymphocytes counts, as well as their ratio, provide the most promising data for the correlation between the COVID-19 course and FMN supplementation. Neutrophils decreased in both cohorts, but only in the experimental group was this decrease statistically significant in the Mann–Whitney *U* test [67.00 (63.00; 73.00) to 63.00 (57.00; 69.00), *p* = 0.0406 vs. 69.00 (58.75; 72.25) to 66.00 (58.50; 71.50), *p* = 0.7904]. Lymphocytes also increased in both groups, but statistical significance has been found for the FMN group only [21.00 (19.00; 27.00) to 27.00 (20.00; 33.00), *p* = 0.0262 vs. 21.50 (19.00; 30.25) to 24.00 (19.00; 31.50), *p* = 0.5651]. Finally, the NLR demonstrated a similar pattern for the experimental and control groups [3.190 (2.267; 3.789) to 2.296 (1.781; 3.190), *p* = 0.0152 vs. 3.117 (1.935; 3.798) to 2.680 (1.888; 3.789), *p* = 0.6224].

**TABLE 3:** Dynamics of main blood and biochemical parameters within 1 week after admission to the hospital in FMN group (*n* = 50) and control group (*n* = 69).

	day 1	day 7	p^a^	p^b^
RBC count, 10^12^ cells per liter, median [IQR]; normal range is 4.0–5.1×10^12^ cells per liter for men and 3.7–4.7×10^12^ cells per liter for women				
FMN group	4.500 [4.210; 4.840]	4.380 [3.950; 4.630]	**0.0406**	**0.0013**
Control group	4.425 [3.865; 4.778]	4.460 [3.880; 4.840]	0.7560	0.6420
p^a^	0.1088	0.3401		
Platelets count, 10^9^ cells per liter, median [IQR]; normal range is 150–450×10^9^ cells per liter				
FMN group	224.0 [182.0; 284]	275.0 [207.0; 341.0]	**0.0363**	**0.0061**
Control group	244.0 [195.0; 300.0]	249.5 [210.5; 309.0]	0.3094	0.2321
p^a^	0.3621	0.4723		
WBC count, 10^9^ cells per liter, median [IQR]; normal range is 4.5–11×10^9^ cells per liter				
FMN group	6.60 [5.20; 8.20]	6.50 [5.00; 8.60]	0.8848	0.7268
Control group	6.40 [5.30; 8.70]	6.00 [5.10; 7.68]	0.2858	0.1357
p^a^	0.8491	0.4481		
Neutrophils ratio, % to WBC count, median [IQR]; normal range is 40–60% to WBC count				
FMN group	67.00 [63.00; 73.00]	63.00 [57.00; 69.00]	**0.0152**	**0.0105**
Control group	69.00 [58.75; 72.25]	66.00 [58.50; 71.50]	0.7904	0.4503
p^a^	0.4838	0.1404		
Lymphocytes ratio, % to WBC count, median [IQR]; normal range is 20–40% to WBC count				
FMN group	21.00 [19.00; 27.00]	27.00 [20.00; 33.00]	**0.0262**	**0.0092**
Control group	21.50 [19.00; 30.25]	24.00 [19.00; 31.50]	0.5651	0.3241
p^a^	0.6461	0.2537		
Monocytes ratio, % to WBC count, median [IQR]; normal range is 3–11% to WBC count				
FMN group	7.0 [5.0; 10.0]	8.0 [5.0; 10.0]	0.8307	0.9225
Control group	8.0 [6.0; 10.0]	7.0 [6.0; 9.0]	0.2713	0.1522
p^a^	0.5542	0.4353		
Neutrophil to lymphocytes ratio, median [IQR]; normal range is ∼1–3				
FMN group	3.190 [2.267; 3.789]	2.296 [1.781; 3.190]	**0.0108**	**0.0242**
Control group	3.117 [1.935; 3.798]	2.680 [1.888; 3.789]	0.6224	0.7777
p^a^	0.6035	0.1729		
Hemoglobin, grams per liter, median [IQR]; normal range is 135–175 g per liter for men, 120–155 g per liter for women				
FMN group	134.0 [120.0; 145.0]	124.0 [118.0; 134.0]	**0.0420**	**0.0010**
Control group	128.5 [117.3; 138.8]	128.0 [117.0; 137.5]	0.7611	0.4918
p^a^	0.2826	0.4439		
C-reactive protein, milligrams per liter, median [IQR]				
FMN group	18.20 [2.570; 64.24]	6.600 [2.003; 39.76]	0.2085	0.0725
Control group	14.21 [3.285; 37.11]	6.530 [1.080; 26.55]	0.1053	0.0798
p^a^	0.6437	0.7406		

a Mann–Whitney *U* test (non-paired, non-parametric, two-tailed).

b Wilcoxon matched-pairs signed rank test (paired, non-parametric, two-tailed).

Bold is for *p* < 0.05.

Surprisingly, we have found a statistically significant increase in the platelets count [224.0 (182.0; 284) to 275.0 (207.0; 341.0), *p* = 0.0363] and a statistically significant decrease in the RBC count [4.500 (4.210; 4.840) to 4.380 (3.950; 4.630), *p* = 0.0406] and hemoglobin value [134.0 (120.0; 145.0) to 124.0 (118.0; 134.0), *p* = 0.0420]. None of these parameters changed significantly in the control group and none of them correlated with COVID-19 severity in our cohort. Although thrombocytopenia (platelets count <150×10^9^ cells per liter) is one of the COVID-19 symptoms (Mei et al., 2020; Zong et al., 2021), we had only seven patients that demonstrated thrombocytopenia within the COVID-19 course. Therefore, we split the normal platelets range (150–450×10^9^ cells per liter) into two parts and evaluated groups with lower (<300×10^9^ cells per liter) and higher (>300×10^9^ cells per liter) platelet counts separately (Supplementary Data S1). In fact, platelets in the lower (<300×10^9^ platelets per liter) FMN group increased from 205.5 [178.5; 240.3] to 272.0 [197.3; 327.3], *p* = 0.0014, while in the higher (>300×10^9^ platelets per liter) FMN group they decreased from 336.0 [325.0; 353.0] to 291.0 [217.0; 352.0], *p* = 0.1657), so median values shifted to the center of normal range.

Moreover, we found a decrease in both hemoglobin and RBC count in the experimental group, but not in the control group. Since hemoglobin and RBC values did not correlate with the severity of COVID-19 (CRP values or lung involvement), we assume that this decrease could be explained with fractional eryptosis occurring via visible light irradiation of the patients’ skin. It is known that the light illumination of red blood cells incubated with riboflavin results in partial cell death (Qadri et al., 2017). Fortunately, both median RBC and hemoglobin changed slightly in absolute values and generally remained in the normal range (Supplementary Data S2). Moreover, riboflavin plays an important role in erythropoiesis, as it improves iron absorption and ferritin mobilization (Suwannasom et al., 2020), so we can assume an increase in the RBC count in the FMN group soon.

We additionally picked out patients with advanced severity of COVID-19 (CRP value >10 mg/l and/or CT grade 2–4 at any time of treatment) and evaluated the median values of all studied biochemical parameters in this sub-cohort (Table 4). The obtained data were similar to Table 3 with statistically significant changes for RBC, platelets, neutrophils, lymphocytes, NLR, and hemoglobin in the experimental group, but not in the control group. However, we additionally found statistically significant differences between the experimental and control groups on day 7 for neutrophils and NLR. On day 1 median values of neutrophils were 68.00 [63.00; 79.00] in the experimental group and 70.00 [63.75; 76.00] in the control group (*p* = 0.7504), while on day 7 median values were 64.00 [55.00; 71.00] and 68.00 [63.00; 77.00], respectively (*p* = 0.0317). Similarly, median NLR on day 1 was 3.342 [2.222; 4.139] in the experimental group and 3.600 [2.680; 4.676] in the control group (*p* = 0.2834), while on day 7 median NLR was 2.240 [1.610; 3.300] and 3.000 [2.207; 4.588], respectively (*p* = 0.0284). We assume this result is direct evidence of the anti-inflammatory efficacy of high doses of riboflavin in COVID-19 patients.

**TABLE 4:** Dynamics of main blood and biochemical parameters within 1 week after admission to the hospital for patients demonstrated CRP value >10 mg/l and/or CT grade 2–4 at any time of treatment in FMN group (*n* = 38) and control group (*n* = 43).

	day 1	day 7	p^a^	p^b^
RBC count, 10^12^ cells per liter, median [IQR]; normal range is 4.0–5.1×10^12^ cells per liter for men and 3.7–4.7×10^12^ cells per liter for women)				
FMN group	4.560 [4.200; 4.860]	4.380 [3.680; 4.620]	0.0352	0.0006
Control group	4.290 [3.710; 4.790]	4.410 [3.805; 4.795]	0.7706	0.4125
p^a^	0.1088	0.5086		
Platelets count, 10^9^ cells per liter, median [IQR]; normal range is 150–450×10^9^ cells per liter				
FMN group	242.0 [203.0; 325.0	291.0 [217.0; 367.0]	**0.0251**	**0.0120**
Control group	229.0 [181.0; 298.0]	248.0 [197.5; 331.0]	0.1522	**0.0312**
p^a^	0.4339	0.2057		
WBC count, 10^9^ cells per liter, median [IQR]; normal range is 4.5–11×10^9^ cells per liter				
FMN group	6.500 [5.200; 8.400]	6.700 [5.100; 8.800]	0.7637	0.8358
Control group	6.900 [5.400; 8.500]	6.100 [4.600; 8.400]	0.3018	0.3252
p^a^	0.6583	0.3305		
Neutrophils ratio, % to WBC count, median [IQR]; normal range is 40–60% to WBC count				
FMN group	67.00 [62.00; 77.50]	63.50 [53.75; 70.25]	**0.0223**	**0.0117**
Control group	70.50 [64.25; 78.00]	68.00 [63.00; 77.00]	0.3938	0.7219
p^a^	0.4295	**0.0155**		
Lymphocytes ratio, % to WBC count, median [IQR]; normal range is 20–40% to WBC count				
FMN group	21.00 [18.00; 27.25]	26.50 [19.75; 34.00]	**0.0335**	**0.0091**
Control group	19.50 [17.00; 24.75]	23.00 [17.00; 29.00]	0.2750	0.3150
p^a^	0.3035	0.0602		
Monocytes ratio, % to WBC count, median [IQR]; normal range is 3–11% to WBC count				
FMN group	7.0 [5.0; 10.0]	7.0 [5.0; 10.0]	0.9368	0.9764
Control group	8.0 [6.0; 11.0]	7.0 [5.0; 8.0]	0.1211	0.1124
p^a^	0.4949	0.3355		
Neutrophil to lymphocytes ratio, median [IQR]; normal range is ∼1–3				
FMN group	3.342 [2.222; 4.139]	2.240 [1.610; 3.300]	**0.0157**	**0.0307**
Control group	3.600 [2.680; 4.676]	3.000 [2.207; 4.588]	0.2777	0.6177
p^a^	0.2834	**0.0284**		
Hemoglobin, grams per liter, median [IQR]; normal range is 135–175 g per liter for men, 120–155 g per liter for women				
FMN group	132.0 [120.0; 145.0]	123.0 [116.0; 134.0]	**0.0356**	**0.0001**
Control group	128.0 [115.0; 135.0]	126.0 [110.0; 139.5]	0.9982	0.6957
p^a^	0.1702	0.4855		
C-reactive protein, milligrams per liter, median [IQR]				
FMN group	26.80 [13.30; 67.90]	16.53 [3.375; 49.43]	0.0638	0.0587
Control group	26.50 [13.83; 63.98]	19.60 [4.030; 50.95]	0.1288	0.1901
p^a^	0.9056	0.6665		

a Mann–Whitney *U*-test (non-paired, non-parametric, two-tailed).

b Wilcoxon matched-pairs signed rank test (paired, non-parametric, two-tailed).

Bold is for *p* < 0.05.

### Correlation of Blood and Biochemical Parameters With ICD-10 Diagnosis

Mental disorders vary greatly in their characteristics, including behavior, pathogenesis, and even biochemical markers (García-Gutiérrez et al., 2020). Based on this knowledge, we can assume alterations in the course of the disease in patients with different psychiatric diagnoses. Our cohort included two main types of diagnosis, namely organic mental spectrum disorders (F0, *n* = 41) and schizophrenia spectrum disorders (F2, *n* = 65), while affective disorders (F3, *n* = 11) and oligophrenia (F7, *n* = 2) were minor fractions. Here we compared main blood and biochemical parameters in patients with organic mental disorders and schizophrenia. The median values are summarized in Table 5. Indeed, F0 patients demonstrated higher inflammation than F2 patients, which was expressed in higher neutrophils [72.00 (66.00; 79.25) vs. 67.00 (60.75; 72.00), *p* = 0.0026], lower lymphocytes [19.00 (13.50; 25.25) vs. 23.35 (19.00; 31.00), *p* = 0.0029], and a higher NLR ratio [3.642 (2.703; 5.923) vs. 2.867 (2.006; 3.640), 0.0020]. In addition, the median CRP value was also higher in the F0 subgroup [24.30 (5.750; 63.14) vs. 16.20 (1.900; 40.74)], although this difference was not significant (*p* = 0.2111). Higher inflammation in the F0 subgroup can be explained by background neuroinflammation, which is one of the central mechanisms in dementia or Alzheimer’s disease (Kinney et al., 2018; Hur et al., 2020). However, schizophrenia is also often discussed in terms of inflammatory biomarkers (Müller et al., 2015; Miller and Goldsmith, 2019), so this issue needs further evaluation. Additionally, median RBC value was lower in F0 subgroup in comparison to F2 [4.320 (3.620; 4.640) vs. 4.540 (4.218; 4.860), 0.0072]. Anemia conditions are often found in chronic psychiatry patients (Korkmaz et al., 2015), so we can suppose mild anemia in F0 subgroup. The other possible explanation is statistical bias since absolute values are rather close.

**TABLE 5:** Main blood and biochemical parameters in organic mental spectrum disorders (F0, n = 41) and schizophrenia spectrum disorders (F2, n = 65) subgroups, median [IQR].

	F0	F2	p^a^
RBC, 10^12^ cells per liter	4.320 [3.620; 4.640]	4.540 [4.218; 4.860]	**0.0072**
Platelets, 10^9^ cells per liter	249.0 [181.0; 298.0]	238.0 [198.8; 304.8]	0.7534
WBC, 10^9^ cells per liter	7.100 [5.600; 8.400]	6.050 [4.900; 8.225]	0.1478
Neutrophils ratio, % to WBC	72.00 [66.00; 79.25]	67.00 [60.75; 72.00]	**0.0026**
Lymphocytes ratio, % to WBC	19.00 [13.50; 25.25]	23.35 [19.00; 31.00]	**0.0029**
Monocytes ratio, % to WBC	7.500 [5.750; 10.00]	7.000 [5.000; 10.00]	0.6559
Neutrophil to lymphocytes ratio	3.642 [2.703; 5.923]	2.867 [2.006; 3.640]	**0.0020**
Hemoglobin, grams per liter	128.0 [111.0; 138.0]	131.0 [120.8; 142.5]	0.2070
C-reactive protein, milligrams per liter	24.30 [5.750; 63.14]	16.20 [1.900; 40.74]	0.2111

a Mann–Whitney *U* test (non-paired, non-parametric, two-tailed).

Bold is for *p* < 0.05.

### Cytokines

A panel of cytokines, including IL-1β, IL-2, IL-6, TNF-α, МРС-1, and IFN-*γ*, was also monitored over the hospital stay for a limited number of patients that were recruited to the trial at a late stage (July 2020). All of these cytokines have been earlier studied for clinical relevance in COVID-19. E.g., IL-6 has been previously shown as a predictor for respiratory failure (Jøntvedt Jørgensen et al., 2020) and disease outcome (Del Valle et al., 2020). TNF-α serum levels have been described as an independent and significant predictor of disease severity and death (Del Valle et al., 2020). Studies involving a larger cohort of severe COVID-19 patients showed the importance of IL-2 levels (Shi et al., 2020), while IL-1β has been demonstrated as a low predictive value marker due to its minor expression in patients (Del Valle et al., 2020). In the current research, IL-6 measurements showed the most promising results, decreasing in the experimental group and growing in control group (Table 6). The limited number of patients with cytokine data resulted in a lack of statistical significance, but in line with absolute values changes, we assume IL-6 as the most interesting marker for further research; TNF-α, МСP-1, and МСP-1 as of moderate interest; and IL-2 and IL1β as of low interest.

**TABLE 6:** Dynamics of cytokines within 1 week after admission to the hospital in FMN group (*n* = 30) and control group (*n* = 14).

	day 1	day 7	p^a^	p^b^
IL-6				
FMN group	8.660 [4.470; 17.00]	5.010 [1.635; 12.63]	0.2686	0.1698
Control group	6.170 [4.433; 16.90]	8.180 [4.570; 11.98]	0.9549	0.8552
p^a^	0.8017	0.5482		
IL-2				
FMN group	8.200 [7.680; 9.370]	7.960 [7.390; 8.930]	0.3217	0.1671
Control group	7.560 [6.870; 8.603]	7.475 [7.110; 9.740]	0.8293	0.9863
p^a^	0.1574	0.5696		
TNF-α				
FMN group	9.440 [5.248; 15.02]	8.520 [4.910; 13.15]	0.6408	0.6661
Control group	11.05 [8.293; 16.20]	8.755 [3.910; 15.49]	0.2136	0.2412
p^a^	0.3023	0.8566		
МСP-1				
FMN group	102.1 [62.96; 141.1]	81.81 [62.18; 106.8]	0.2523	0.2801
Control group	69.91 [61.55; 163.3]	73.11 [60.48; 99.23]	0.6434	0.7354
p^a^	0.4811	0.4810		
IL1β				
FMN group	1.500 [0.870; 2.040]	1.480 [0.690; 1.860]	0.7023	0.5598
Control group	1.185 [0.870; 2.140]	1.050 [0.668; 1.525]	0.4058	0.3916
p^a^	0.7186	0.2718		
IFN-*γ*				
FMN group	4.610 [2.060; 8.210]	5.570 [2.970; 8.290]	0.7475	0.6710
Control group	3.550 [1.325; 6.485]	4.020 [2.280; 5.390]	0.5633	>0.9999
p^a^	0.2011	0.0978		

a Mann–Whitney *U* test (non-paired, non-parametric, two-tailed).

b Wilcoxon matched-pairs signed rank test (paired, non-parametric, two-tailed).

## Conclusion

An observational trial of riboflavin (vitamin B2) impact on the immune status of COVID-19 patients with mental disorders has been performed. We demonstrated that a full course of riboflavin supplementation (10 mg of flavin mononucleotide intramuscularly twice a day within 7 days) correlated with a normalization of clinically relevant immune markers (neutrophils and lymphocytes counts, as well as their ratio) in COVID-19 patients. We also found that patients with organic disorders (F0 in ICD-10) demonstrated higher inflammation than patients with schizophrenia patients (F2 in ICD-10). We suppose that riboflavin supplementation could be promising for decreasing inflammation in COVID-19, and further evaluation is required.

## Data Availability

The original contributions presented in the study are included in the article/Supplementary Material, further inquiries can be directed to the corresponding authors.
