# Supplementary material for: Riboflavin for COVID-19 Adjuvant Treatment in Patients With Mental Health Disorders: Observational Study
Source: Front Pharmacol. 2022 Mar 10;13:755745. doi: 10.3389/fphar.2022.755745 (PMC8960625; doi:10.3389/fphar.2022.755745)
Supplement: Supplementary file 1 [file DataSheet1.docx]

Supplementary data





**Supplementary data 1.** Median value of platelets within one week after admission to the hospital in sub-groups with lower (< 300×10^9^ cells per liter) and higher (> 300×10^9^ cells per liter) platelets at day 1. Box and whisker plot with Tukey modification, the borders of normal range (150-450×10^9^ cells per liter) are shown with dotted line.


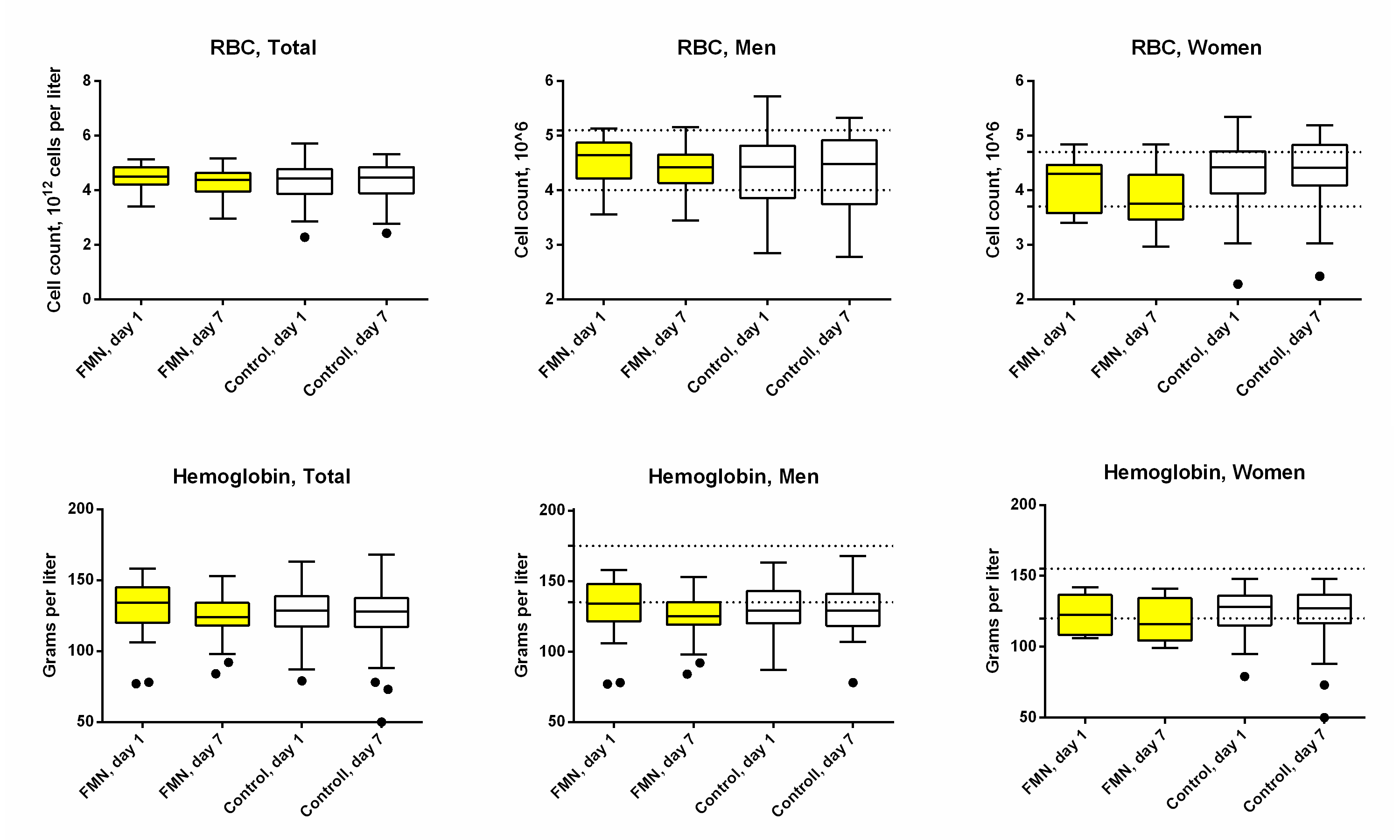


**Supplementary data 2.** Median value of RBC count and hemoglobin within one week after admission to the hospital. Box and whisker plot with Tukey modification, the borders of normal range for each gender are shown with dotted line.
